# Diagnostic and prognostic values of upregulated SPC25 in patients with hepatocellular carcinoma

**DOI:** 10.7717/peerj.9535

**Published:** 2020-07-16

**Authors:** Xiaolin Yang, Hongzhi Sun, Ying Song, Li Yang, Haibo Liu

**Affiliations:** 1 Department of Geriatrics, the First Hospital of Jilin University, Changchun, China; 2 Department of Emergency and Critical Care Medicine, The Second Hospital of Jilin University, Changchun, China; 3 Department of Gastroenterology, The Second Hospital of Jilin University, Changchun, China; 4 Department of Gastroenterology, First Automobile Works General Hospital of Jilin Province, Changchun, China; 5 Department of Emergency, The First Hospital of Jilin University, Changchun, China

**Keywords:** Hepatocellular carcinoma, Biomarker, TCGA, SPC25, Diagnosis, Prognosis

## Abstract

**Background:**

Spindle pole body component 25 (SPC25) plays a vital role in many cellular processes, such as tumorigenesis. However, the clinical significance of SPC25 in hepatocellular carcinoma (HCC) has not been investigated. This study aimed to explore the expression patterns of SPC25 in HCC and non-neoplastic tissues and to investigate the diagnostic and prognostic values of SPC25.

**Method:**

The expression of SPC25 was examined in 374 HCC issues and 50 non-neoplastic tissues from The Cancer Genome Atlas (TCGA) cohort. The diagnostic and prognostic values of SPC25 were analyzed via receiver operating characteristic (ROC) curve and survival analyses, respectively. Univariate and multivariate Cox regression analyses were used to identify the prognostic factors and to establish a nomogram. The diagnostic and prognostic values were further validated in an external cohort from the International Cancer Genome Consortium (ICGC) database.

**Results:**

The expression of SPC25 in HCC tissues was significantly higher than that in normal tissues in both cohorts (all *P* < 0.001). The ROC curve analysis indicated that SPC25 expression has high diagnostic value in HCC with area under the curve (AUC) value of 0.969 (95% confidence interval [CI] [0.948–0.984]) and 0.945 (95% CI [0.920–0.965]) for TCGA and ICGC cohorts, respectively. Patients with HCC exhibiting high SPC25 expression were associated with worse prognosis than those exhibiting low SPC25 expression in both cohorts (all *P* < 0.001). SPC25 was independently associated with overall survival in both cohorts (all *P* < 0.001). The concordance indices of the nomogram for predicting overall survival in TCGA and ICGC cohorts were 0.647 and 0.805, respectively, which were higher than those of the American Joint Committee on Cancer (AJCC) staging system.

**Conclusion:**

SPC25 was upregulated in HCC and independently predicted poor overall survival of patients with HCC. Therefore, SPC25 is an effective diagnostic and prognostic biomarker for HCC. An SPC25-based nomogram was more accurate and useful than the AJCC staging system to predict prognosis of HCC.

## Introduction

Hepatocellular carcinoma (HCC) is the predominant cancer type among the primary liver cancers. Among the malignant tumors, HCC is the sixth most common cancer and is associated with fourth highest cancer-related mortality (Bray et al., 2018). In 2008, approximately 840,000 new cases of HCC were recorded globally, which resulted in approximately 780,000 deaths (Bray et al., 2018). In China, more than 466,100 new HCC cases and 422,100 HCC-related deaths were reported in 2015 (Chen et al., 2016). Nowadays, rapid advances has been made in the therapy of HCC thanks to the encouraging progress in early diagnosis and cancer therapeutic methods such as imaging techniques, liver transplantation, surgical resection, local ablation, and transcatheter arterial chemoembolization and comprehensive therapy (Zhang et al., 2018). However, the 5-year survival rate of patients with HCC remains low due to the high rates of local recurrence and distant metastasis (Allemani et al., 2015). Several studies have demonstrated that various aberrantly expressed genes can be used to determine HCC prognosis (Chen et al., 2018b; Feng et al., 2019; Yu et al., 2019; Zhu et al., 2019). For example, low expression of CTC-297N7.9 is associated with poor prognosis in HCC patients, and could be used as an independent prognostic indicator in HCC patients. However, its diagnostic capability is limited in discriminating HCC tissues from normal tissues with an AUC of 0.74 (Zhu et al., 2019). The knockdown of TMEM16A inhibited HCC cell proliferation, migration and induced cell apoptosis, and upregulation of TMEM16A induced HCC cell growth, migration and reduced cell apoptosis (Zhang et al., 2020). However, its diagnostic capability in discriminating HCC tissues from normal tissues was unknown. Currently, serum alpha-fetoprotein is the most reliable HCC biomarker although it has low sensitivity and specificity in the diagnosis of HCC (Chaiteerakij, Addissie & Roberts, 2015; Daniele et al., 2004). Therefore, there is an urgent need to identify and validate novel reliable diagnostic and prognostic biomarkers for HCC.

Spindle pole body component 25 (SPC25), a component of the nuclear division cycle 80 (Ndc80) complex, is involved in kinetochore-microtubule interactions and spindle checkpoint activity (McCleland et al., 2004; Tooley & Stukenberg, 2011). Previous studies have reported that dysregulated SPC25 expression is associated with the oncogenic process and malignant phenotypes of several cancers. The upregulated expression of SPC25 has been reported in colorectal, gastric cancers, breast, and lung adenocarcinoma. Additionally, SPC25 is involved in carcinogenesis, cancer cell growth, and metastasis (Chen et al., 2018a; Jeong et al., 2018; Kaneko et al., 2009; Wang et al., 2019). A cell-based assay revealed that the SPC25 can be a potential biomarker for Alzheimer’s disease as the expression level of SPC25 was significantly upregulated in the serum samples of patients with mild cognitive impairment (Zhang et al., 2018). In the HCC cells, PRC1 regulates the expression and function of recurrence-associated genes, such as SPC25, KIF11 FANCI, and KIF23 via Wnt signalling (Zhu et al., 2019). However, the correlation between SPC25 expression and HCC for diagnosis and prognosis of HCC has not been previously evaluated. This study aimed to analyze the SPC25 mRNA expression patterns and to determine the diagnostic and prognostic values of SPC25 expression in HCC.

In his study, we evaluated the mRNA expression of SPC25 in patients with HCC based on the data obtained from The Cancer Genome Atlas (TCGA) database. Additionally, the correlation between SPC25 expression and clinicopathological features, as well as the potential diagnostic and prognostic values of SPC25 in patients with HCC were analyzed. Furthermore, TCGA analysis results were validated using an external cohort from the International Cancer Genome Consortium (ICGC) database. A nomogram prognostic model based on SPC25 was constructed. The accuracy of the prognostic model was compared with that of the currently used American Joint Committee on Cancer (AJCC) staging system in both cohorts.

## Material and Methods

### SPC25 expression data and clinicopathological characteristics

The gene expression profiles of 374 human HCC tissues and 50 non-neoplastic tissues, as well as the clinical data of patients, such as age at diagnosis, sex, histologic grade, pathological stage, vascular invasion, family history of cancer, survival status, and survival time were downloaded from TCGA Liver Hepatocellular Carcinoma (TCGA-LIHC; https://portal.gdc.cancer.gov/) database. The mRNA expression levels of SPC25 in TCGA cohort were extracted. The expression levels of SPC25 in HCC and non-tumorous tissues were comparatively analyzed in R (version 3.6.3) using the limma package (Ritchie et al., 2015). Next, the expression data and clinical data were merged using the patients’ unique identification numbers. In total, 370 patients were included in the final analysis. The relationship between SPC25 expression and clinical parameters was analyzed in these patients. The patients were classified into high expression and low expression groups based on the median SPC25 expression level in patients with HCC. The differences in the overall survival rates between the two groups were analyzed using R (version 3.6.3) ‘survival’ package (https://CRAN.R-project.org/package=survival). Furthermore, ESTIMATE (Estimation of Stromal and Immune cells in Malignant Tumor tissues using Expression data) algorithm was used to estimate the tumor purity, and the presence of infiltrating stromal/immune cells in tumor microenvironment for each sample. We analyzed the relationships between SPC25 expression and immune cell infiltration (Yoshihara et al., 2013).

### Statistical analysis

The correlation between SPC25 expression and clinicopathological characteristics was evaluated using the Chi-square test and logistic regression and was represented with box plots. The area under the curve (AUC) value obtained from the receiver operating characteristic (ROC) curve analysis was used to determine the diagnostic effectiveness of SPC25 in discriminating HCC tissues from normal tissues in both cohorts using the pROC package (Robin et al., 2011). An AUC value greater than 0.85 is considered to have outstanding predictive value (Metz, 1978). Kaplan–Meier curves with *P*-value calculated by the log-rank test were used to compare the differences in overall survival rates. The univariate Cox regression analysis was performed to identify the possible prognostic variables. The multivariate Cox analysis was performed to verify the effect of SPC25 expression level on prognosis along with other clinical factors. The variables with *P* < 0.05 in the univariate analysis were adjusted in the multivariate analysis. A nomogram was established based on the independent clinicopathological factors in TCGA cohort using the ‘rms’ package (https://CRAN.R-project.org/package=rms). The nomogram was validated by analyzing discrimination and calibration curves in both cohorts. The concordance index (C-index) was used to evaluate the discrimination of the nomogram. The decision curve analysis (DCA, https://CRAN.R-project.org/package=rmda) was performed to test the clinical utility of the model between the SPC25-based nomogram, the AJCC staging system, and alpha-fetoprotein levels. The differences were considered statistically significant when the *P*-value was less than 0.05. All statistical analyses were performed using the R software (V.3.6.3, http://www.r-project.org).

### Validation of the diagnostic and prognostic values of SPC25 using an external database

To obtain reliable results, the mRNA expression profiles of 240 HCC cases and 260 adjacent nontumor tissues with clinical data downloaded from the ICGC database (https://dcc.icgc.org/projects/LIRI-JP) were analyzed following the same methodology used to analyze TCGA cohort. Among the ICGC cohort, 202 adjacent nontumor tissues from patients with cancer and 240 primary tumor samples from patients with HCC were included in this study to validate the results of TCGA cohort analysis.

## Results

### SPC25 expression in patients with varied clinicopathological parameters

**Figure 1:**
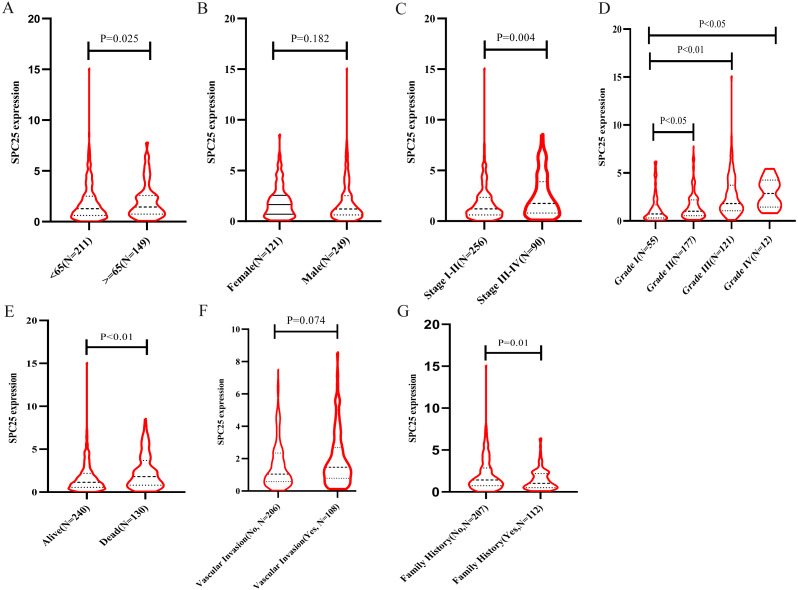
SPC25 mRNA expression varied significantly in patients with hepatocellular carcinoma (HCC) exhibiting different clinicopathological characteristics. The SPC25 expression level was compared between HCC samples and non-tumorous samples in from The Cancer Genome Atlas (TCGA) cohort according to (A) age, (B) gender, (C) pathological stage, (D) histologic grade, (E) survival status, (F) vascular invasion, and (G) family history of cancer.

**Figure 2:**
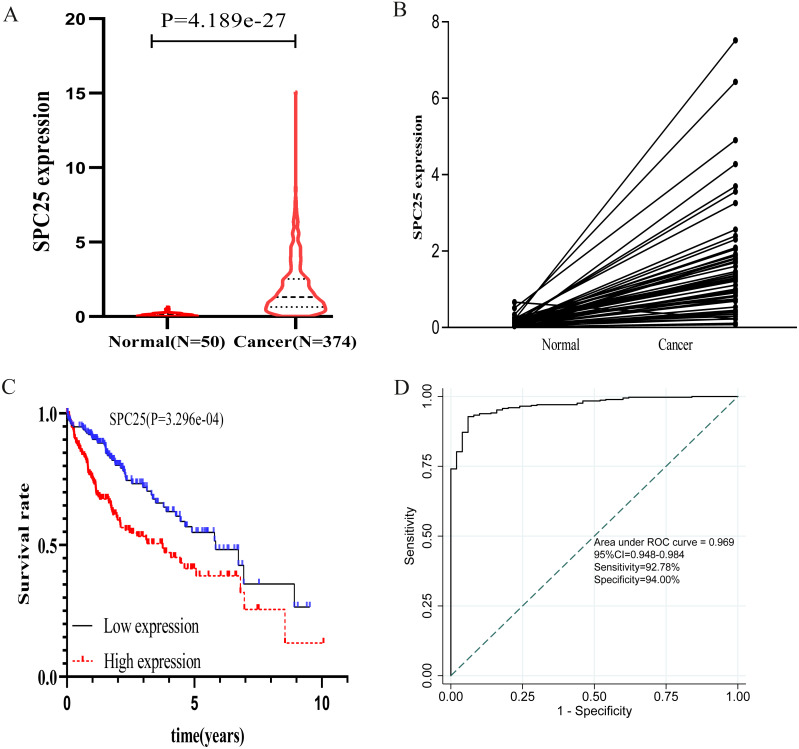
The expression levels and diagnostic and prognostic values of SPC25 in hepatocellular carcinoma. (A) The expression level of SPC25 in hepatocellular carcinoma tissues was significantly higher than that in non-tumorous tissues (*P* < 0.0001). (B) In 50 paired HCC and non-tumorous tissues, the expression level of SPC25 in hepatocellular carcinoma tissues was upregulated when compared with that in adjacent non-tumorous tissues (*P* < 0.0001). (C) Impact of SPC25 expression on overall survival in patients with hepatocellular carcinoma in The Cancer Genome Atlas (TCGA) cohort. (D) Diagnosis value of SPC25 expression in discriminating hepatocellular carcinoma tissues from non-tumorous tissues.

The correlation between SPC25 expression and clinicopathological characteristics in patients with HCC was represented using the box plots. The clinical data of patients in the internal validation group and the external validation group were shown in Table 1. As shown in Fig. 1, high SPC25 expression was associated with patient age (*P* = 0.025, Fig. 1A), pathological stage (*P* = 0.004, Fig. 1C), histologic grade (*P* < 0.0001, Fig. 1D), survival status (*P* = 0.00029, Fig. 1E), and family history of cancer (*P* = 0.01, Fig. 1G). However, SPC25 expression was not significantly correlated with sex and vascular invasion (all *P* > 0.05, Figs. 1B and 1F). The differences in the SPC25 expression level in HCC tissues and non-tumorous tissues were represented using a boxplot. The expression level of SPC25 in HCC tissues was significantly upregulated when compared with that in normal tissues (Fig. 2A, *P* < 0.0001). Next, the expression level of SPC25 in 50 paired HCC tissues and non-neoplastic tissues from TCGA cohort was analyzed. The expression level of SPC25 in HCC tissues was upregulated when compared with that in non-neoplastic tissues (Fig. 2B, *P* < 0.0001).

**Table 1:** Clinical data of patients in the internal validation group and the external validation group.

**Variables**	**Subgroups**	**TCGA**	**ICGC**
Age			
	<65	221	85
	≥65	149	155
Sex			
	Male	249	179
	Female	121	61
Stage			
	I	171	36
	II	85	109
	III	85	74
	IV	5	21
	NA	24	0
Grade			
	I	55	–
	II	177	–
	III	121	–
	IV	12	–
	NA	5	–
Survival status			
	Dead	130	43
	Living	240	197
Vascular invasion			
	Positive	108	–
	Negative	206	–
	NA	56	–
Family history			
	Positive	112	76
	Negative	207	149
	NA	51	15
Prior malignancy			
	Positive	–	32
	Negative	–	208
	NA	–	0

Logistic regression analysis revealed that upregulated SPC25 expression in HCC was closely associated with age (odds risk [OR] = 0.568 for ≥65 vs <65, 95% CI [0.372–0.863], *P* = 0.0083), pathological stage (OR = 1.957 for stage III + stage IV vs stage I + stage II, 95% CI [1.202–3.223], *P* = 0.0075), histologic grade (OR= 4.936 for grade 3 vs grade 1, 95% CI [2.506–10.104], *P* < 0.001; OR= 12.187 for grade 4 vs grade 1, 95% CI [2.827–85.344], *P* = 0.0026), and patients survival status (OR = 1.958 for dead vs. alive, 95% CI [1.272–3.036], *P* = 0.0024) (Table 2).

### Diagnostic and prognostic values of upregulated SPC25 expression in patients with HCC

The prognostic value of SPC25 in patients with HCC was evaluated. The Kaplan–Meier curve demonstrated that high SPC25 expression was significantly associated with unfavorable overall survival (Fig. 2C, *P* < 0.001). To assess the diagnostic value of SPC25, we generated an ROC curve using the SPC25 expression data from 370 HCC tissues and 50 non-neoplastic tissues. As shown in Fig. 2D, SPC25 had an excellent diagnostic value in discriminating HCC tissues from normal tissues with an AUC of 0.969 (95% CI [0.94.8–0.984]), sensitivity of 92.78% (95% CI [89.7–95.2]%), and specificity of 94.00 (95% CI [83.5–98.7]%).

**Figure 3:**
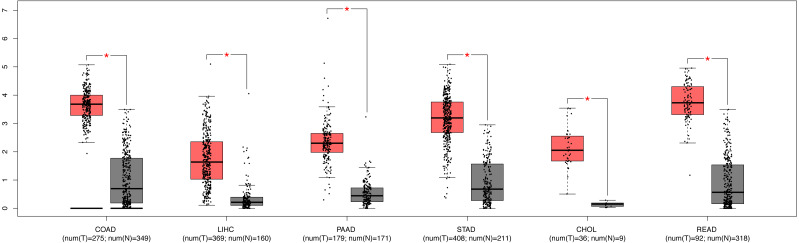
The expression of SPC25 in colon adenocarcinoma, LIHC, pancreatic adenocarcinoma, stomach adenocarcinoma, rectum adenocarcinoma, cholangiocarcinoma, and corresponding non-tumorous tissues.

We further explored the expression level of SPC25 in other gastrointestinal tract malignances. As shown in Fig. 3, the expression levels of SPC25 in cholangiocarcinoma, colon adenocarcinoma, pancreatic adenocarcinoma, rectum adenocarcinoma, LIHC, and stomach adenocarcinoma were significantly higher than those in non-neoplastic tissues. To confirm the prognostic value of SPC25 expression, univariate and multivariate analyses were performed. The univariate analysis revealed that age, sex, pathological stage, and SPC25 expression were significantly correlated with overall survival (all *P* < 0.05). After adjusting for other prognostic variables, the multivariate analysis revealed that SPC25 expression was independently associated with overall survival (hazard ratio [HR] = 1.76, 95% CI [1.100–2.815], *P* = 0.0183). All other independent risk variables, including age and pathological stage, and the corresponding HR and 95% CI are listed in Table 3.

**Table 2:** Logistic regression of SPC25 expression associated with clinical pathological characteristics.

Clinical characteristics	Total (*N*)	Odds ratio in SPC25 expression	95% CI	*P*-Value
Pathological stage (III + IV *vs* I + II)	346	1.957	1.202–3.223	**0.0075**
Histologic grade (G2 *vs* G1)	232	1.792	0.946–3.523	0.0802
(G3 *vs* G1)	176	4.936	2.506–10.104	**<0.0001**
(G4 *vs* G1)	67	12.187	2.827–85.344	**0.0026**
Age (≥65 *vs* <65)	370	0.568	0.372–0.863	**0.0083**
Gender (Male *vs* Female)	370	0.621	0.542–1.127	0.0854
Survival status (Dead *vs* Alive)	370	1.958	1.272–3.036	**0.0024**
Family history of cancer (Yes *vs* No)	319	0.686	0.431–1.088	0.11
Vascular invasion (Yes *vs* No)	314	1.487	0.932–2.383	0.0969

**Notes.**

* Statistically significant *P*-values are given in bold, *P* < 0.05.

**Table 3:** Univariate and multivariate analysis of the relationship of SPC25 expression with overall survival among hepatocellular carcinoma patients.

Variables	Univariate analysis			Multivariate analysis		
	HR	95% CI	*P*-value	HR	HR.95 L	*P*-value
Age	1.021	1.002–1.040	**0.0267**	1.02	1.001–1.040	**0.0421**
Sex	0.587	0.373–0.923	**0.0211**	0.796	0.490–1.294	0.358
Stage	1.465	1.142–1.879	**0.0027**	1.415	1.097–1.824	**0.0075**
SPC25	1.776	1.121–2.814	**0.0144**	1.76	1.100–2.815	**0.0183**
Grade	1.221	0.893–1.669	0.2116			
Vascular invasion	1.443	0.904–2.305	0.1244			
Family history	1.418	0.903–2.225	0.1291			

**Notes.**

SPC25 spindle pole body component 25 HR hazard ratio CI confidence interval

Bold values indicate *P* < 0.05.

### Nomogram construction and validation

**Figure 4:**
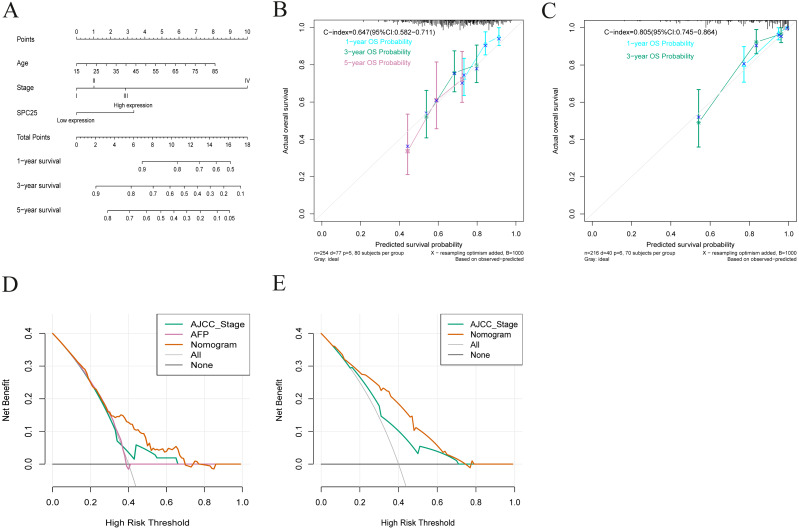
Nomogram for overall survival, calibration plots of the nomogram, and decision curve analysis of nomogram and American Joint Committee on Cancer (AJCC) staging system. (A) The nomogram predicted 1-, 3-, and 5-year overall survival rates of patients with hepatocellular carcinoma in The Cancer Genome Atlas (TCGA) cohort. (B) The calibration curves predict 1-, 3-, and 5-year overall survival rates in TCGA cohort. (C) The calibration curves predicting the 1- and 3-year overall survival in the International Cancer Genome Consortium cohort. (D) The predicted survival rates in TCGA cohort by nomogram and AJCC staging system were comparatively analyzed. (E) The predicted survival rates in the International Cancer Genome Consortium cohort by nomogram and AJCC staging system were comparatively analyzed.

Based on the results from multivariate Cox analysis, age, pathological stage, and SPC25 expression were identified as independent prognostic factors for overall survival. Nomograms for predicting 1-, 3-, and 5- year overall survival rates were established based on these independent risk factors in TCGA cohort (Fig. 4A). The internal validation in TCGA cohort demonstrated that the C-index of nomogram for overall survival was 0.647 (95% CI [0.582–0.711]), which was higher than that of the AJCC staging (C-index = 0.558; 95% CI [0.494–0.622]) and alpha-fetoprotein levels (C-index = 0.425, 95% CI [0.350–0.501]). In the ICGC cohort, the C-index for nomogram to predict overall survival was 0.805 (95% CI [0.745–0.864]), which was higher than that of AJCC staging (C-index = 0.705, 95% CI [0.634–0.780]). The calibration plots in TCGA cohort (Fig. 4B) and ICGC cohort (Fig. 4C) demonstrated an excellent agreement between the nomogram prediction and observed estimates for 1-, 3-, and 5-year overall survival rates. In the DCA analysis, the nomogram presented preferable net benefit with a wider range of threshold probabilities when compared with the AJCC stage system and alpha-fetoprotein levels in both cohorts (Figs. 4D and 4E). These results suggest that the SPC25-based nomogram is superior to the AJCC stage system in predicting HCC prognosis.

### Validation of the diagnostic and prognostic values of SPC25 in the ICGC cohort

**Figure 5:**
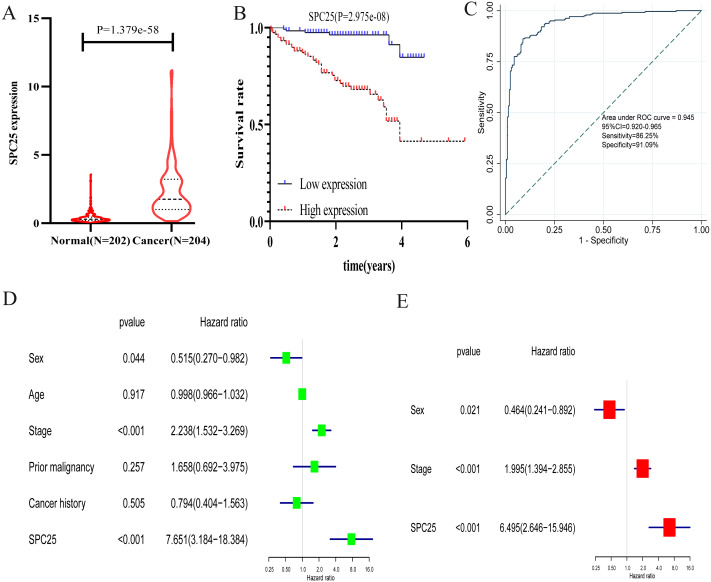
The validation of the expression levels and diagnostic and prognostic values of SPC25 in hepatocellular carcinoma in the International Cancer Genome Consortium cohort. (A) The expression level of SPC25 in hepatocellular carcinoma was upregulated when compared with that in normal solid tissues (*P* < 0.0001). (B) High SPC25 expression was associated with an unfavorable overall survival in patients with hepatocellular carcinoma (*P* < 0.0001). (C) Diagnosis value of SPC25 expression in hepatocellular carcinoma in the International Cancer Genome Consortium cohort. (D) Univariate analysis and (E) multivariate analysis of the correlation of SPC25 expression with overall survival among patients with hepatocellular carcinoma.

External validation further confirmed that the expression of SPC25 in HCC tissues was higher than that in adjacent tissues (Fig. 5A, *P* < 0.0001). The Kaplan-Meir analysis revealed that the high SPC25 expression group had an unfavorable prognosis when compared with the low SPC25 expression group (Fig. 5B, *P* < 0.001). The diagnostic value of SPC25 also demonstrated excellent performance in the ICGC cohort with an AUC of 0.945 (95% CI [0.920–0.965], Fig. 5C). The univariate analysis indicated that SPC25 expression, sex, and pathological stage were correlated with overall survival (all *P* < 0.005, Fig. 5D). After adjusting for other risk factors, the multivariate analysis results confirmed that SPC25 expression was independently correlated with survival in patients with HCC (HR = 6.495, 95% CI [2.646–15.946], *P* < 0.001, Fig. 5E). These results indicate that SPC25 is a novel diagnostic and prognostic biomarker for HCC.

### Correlation of SPC25 with the proportion of tumor-infiltrating immune cells

**Figure 6:**
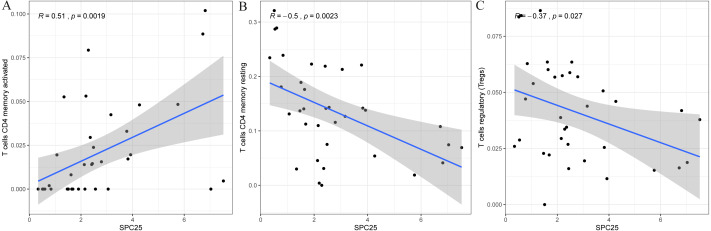
Scatter plot showed the correlation of activated CD4 T memory cells (A), regulatory T cells (B), and resting memory CD4 + T cells (C) tumor-infiltrating immune cells proportion with the SPC25 expression (*P* < 0.05). The blue line in each plot was fitted linear model suggesting the proportion of the immune cell along with SPC25 expression, and Pearson coefficient was used for the correlation test.

Furthermore, the correlation of SPC25 expression with the immune microenvironment was explored among 21 kinds of immune cell profiles in LIHC samples. The results from the correlation analyses demonstrated that a total of 3 kinds of tumor-infiltrating immune cells were correlated with the expression of SPC25 (Fig. 6). Among them, activated CD4 T memory cells were positively correlated with SPC25 expression (Fig. 6A); two kinds of tumor-infiltrating immune cells were negatively correlated with SPC25 expression, including regulatory T cells (Tregs), and resting memory CD4 + T cells (Figs. 6B–6C). These results further indicated that the levels of SPC25 affected the immune activity of immune microenvironment to a certain extent.

## Discussion

In this study, the expression of SPC25 in HCC tissues was upregulated when compared with that in normal tissues. The expression of SPC25 was related to age, pathological stage, histologic grade, survival status, and family history of cancer. The Kaplan–Meier analysis of overall survival revealed that high expression of SPC25 was associated with unfavorable prognosis in patients with HCC. The ROC analysis confirmed that SPC25 had an excellent diagnostic value in discriminating HCC tissues from normal tissues. The univariate and multivariate analyses revealed that upregulated SPC25 expression was an independent prognostic factor of short overall survival. These results were successfully validated in an external (ICGC) cohort. These findings suggest that SPC25 expression can serve as a promising biomarker of poor prognosis in patients with HCC.

SPC25, a key component of the Ndc80 complex, can form a heterodimer with SPC24 to regulate microtubule-kinetochore attachment, chromosome alignment, and spindle checkpoint activation during mitosis process (McCleland et al., 2004; Sun et al., 2010). Recent studies have reported that the aberrant expression of Ndc80 complex is involved in the progression of human cancer (Fu & Shao, 2016; Hu et al., 2015). Tumorigenesis can result from genetic instability in the cell cycle, which is caused due to defects in chromosomal segregation, a process in which kinetochores play a key role (Tooley & Stukenberg, 2011). A previous study revealed that SPC25 expression in the basal breast cancer subtype is markedly upregulated when compared with that in other subtypes and that that enhanced SPC25 expression is related to decreased overall survival (Pathania et al., 2016). SPC25 upregulation can increase cancer stem cell properties in lung adenocarcinoma and independently predict poor survival in patients with lung adenocarcinoma. The knockdown of SPC25 impaired the cancer stem cell properties in lung adenocarcinoma (Chen et al., 2018a). A recent study also revealed that SPC25 knockdown promoted the apoptosis of prostate cancer cells (Cui et al., 2018). These findings demonstrate the potential contribution of SPC25 upregulation to poor survival in HCC.

As the expression of SPC25 plays an important role in overall survival in patients with HCC, we determined whether it can be used to develop an improved prognostic model. A nomogram based on independent prognostic factors was constructed. Compared with the AJCC staging system, the nomogram exhibited an excellent predictive ability in TCGA and ICGC cohorts. Discrimination, calibration, and DCA of the nomogram were verified in the internal and external cohorts. The prognostic nomogram had enhanced performance when compared with the AJCC staging system. Currently, the tumor-node-metastasis (TNM) grading system released by the AJCC is the most commonly used staging system for HCC. However, the efficiency of TNM grading system in prognosis prediction is gradually lost as it is only based on the number of metastatic lymph nodes (Charlton et al., 2014; Wang et al., 2012). In this study, a nomogram was developed based on the SPC25 expression levels. The nomogram provided a more accurate individualized prediction of overall survival in HCC when compared to the AJCC stage system. This was consistent with a previous SEER-based study on prediction of overall survival and cancer-specific survival in patients with HCC (Xiao et al., 2019).

To the best of our knowledge, this is the first study to demonstrate SPC25 as a useful diagnostic biomarker for HCC, as well as a powerful independent prognostic factor for patients with HCC. However, this study has several limitations. This study only explored the prognostic value of SPC25 based only on the dysregulated mRNA level. The dysregulated protein expression level of SPC25 was not verified. Additionally, the clinical information from TCGA database was not comprehensive. Further clinical data should be included to improve the evaluation of correlation between SPC25 expression and HCC. Furthermore, this study was based on bioinformatics analysis. The in silico findings must be validated by *in vivo* and *in vitro* studies to elucidate the function and mechanism of upregulated SPC25 expression in HCC prognosis.

## Conclusion

This study, for the first time, demonstrated that SPC25 expression, which was upregulated in HCC tissues, was correlated with an unfavorable prognosis in patients with HCC using TCGA and ICGC cohorts. SPC25 expression may be a powerful diagnostic and prognostic biomarker for HCC. An SPC25-based nomogram was more accurate and useful than the AJCC staging system.

## Additional Information and Declarations

### Data Availability

The following information was supplied regarding data availability: Data are available at TCGA (search terms: SPC25 and TCGA-LIHC) and at the ICGC Data Portal (search terms: LIHC-JP; https://dcc.icgc.org/).
